# Feasibility of investigating the association between bacterial pathogens and oral leukoplakia in low and middle income countries: A population-based pilot study in India

**DOI:** 10.1371/journal.pone.0251017

**Published:** 2021-04-29

**Authors:** Krithiga Shridhar, Aastha Aggarwal, Ishita Rawal, Ruby Gupta, Shet Masih, Ravi Mehrotra, Theresa W. Gillespie, Preet K. Dhillon, Dominique S. Michaud, Dorairaj Prabhakaran, Michael Goodman

**Affiliations:** 1 Centre for Chronic Conditions and Injuries, Public Health Foundation of India, Gurugram, India; 2 Centre for Chronic Disease Control, New Delhi, India; 3 Molecular Diagnostics Research Laboratory, Chandigarh, India; 4 India Cancer Research Consortium, Indian Council of Medical Research, New Delhi, India; 5 Emory University Rollins School of Public Health, Atlanta, Georgia, United States of America; 6 Division of Surgical Oncology, Department of Surgery, Winship Cancer Institute, Emory University School of Medicine, Atlanta, Georgia, United States of America; 7 Genentech Roche, California, United States of America; 8 Department of Public Health & Community Medicine, Tufts University School of Medicine, Boston, MA, United States of America; 9 London School of Hygiene and Tropical Medicine, London, United Kingdom; University of the Pacific, UNITED STATES

## Abstract

**Background:**

Certain oral bacterial pathogens may play a role in oral carcinogenesis. We assessed the feasibility of conducting a population-based study in India to examine the distributions and levels of *Porphyromonas gingivalis*, *Fusobacterium nucleatum* and *Prevotella intermedia* in relation to oral leukoplakia (a potentially malignant disorder) and other participant characteristics.

**Methods:**

This exploratory case-control study was nested within a large urban Indian cohort and the data included 22 men and women with oral leukoplakia (cases) and 69 leukoplakia-free controls. Each participant provided a salivary rinse sample, and a subset of 34 participants (9 cases; 25 controls) also provided a gingival swab sample from keratinized gingival surface for quantitative polymerase chain reaction (qPCR).

**Results:**

Neither the distribution nor the levels of pathogens were associated with oral leukoplakia; however, individual pathogen levels were more strongly correlated with each other in cases compared to controls. Among controls, the median level of total pathogens was the highest (7.55×10^4^ copies/ng DNA) among persons of low socioeconomic status. Salivary rinse provided better DNA concentration than gingival swab for qPCR analysis (mean concentration: 1.8 ng/μl vs. 0.2 ng/μl).

**Conclusions:**

This study confirms the feasibility of population studies evaluating oral microbiome in low-resource settings and identifies promising leads for future research.

## Introduction

Mechanistic studies of common oral bacterial species [1–3], and epidemiological studies with high-throughput sequencing of oral microbiome [4–7] have identified discrete bacterial genera that may be associated with increased risk of oral cancers, influencing early [5,7–11] and late carcinogenesis [12]. Especially compelling is the evidence that supports the carcinogenic potential of bacterial pathogens such as *Porphyromonas gingivalis*, *Fusobacterium nucleatum* and *Prevotella sp*. [1–5]. These oral bacteria are associated with periodontal disease, a chronic inflammatory condition resulting in loss of soft tissue and bone surrounding the teeth; and are generally present in low levels in saliva, dental plaque, gingival and mucosal surfaces, but overgrow in disease [13]. The balance in composition and levels of pathogens is regulated by oral hygiene, lifestyle factors as well as the genetic landscape of the individuals [13]. There is increasing interest in better understanding the epidemiological patterns and biological mechanisms by which oral bacterial flora may influence the risk of oral, orodigestive tract and other malignancies [14].

About a third of global oral cancer burden falls on India [15], where the disease affects young adults with high prevalence of risk factors such as the use of chewing tobacco and betel quid [16]. While the role of tobacco and betel quid in the etiology of oral malignancies is well documented [17], recent data indicate that certain regions of India also experience increasing incidence of oral cancer, specifically tongue cancer, among persons without identifiable risk factors [17–20]. These observations underscore the importance of evaluating other possible modifiable causes and mechanisms of oral cancer, including oral microbiome that may act independently of, and perhaps in synergy with, established risk factors [1–4,21].

Addressing this issue will require large-scale epidemiological studies assessing the roles of bacterial pathogens in oral neoplasia in high-risk populations. Such populations reside primarily in low and middle income countries (LMICs), where implementation of large-scale studies of this type with may encounter important barriers. To begin assessing the feasibility of such studies we explored the distribution of *P*. *gingivalis*, *F*. *nucleatum* and *P*. *intermedia* in salivary rinse samples of an urban North Indian population using real-time quantitative polymerase chain reaction (qPCR). We examined the proportion of detection and quantification and the levels of these pathogens in relation to clinical oral leukoplakia (a potentially malignant disorder) in a case-control study nested within a large population-based cohort. We also examined the associations of these pathogens with established and emerging risk factors of oral cancer such as sociodemographic characteristics, lifestyle factors and clinically diagnosed periodontal disease in leukoplakia-free participants. In a sub-set of participants, we further explored whether samples of salivary rinse or gingival swab are more suitable for qPCR analysis in population-based studies. The overall goal of the project was to assess the feasibility of carrying out this type of research in the general population of India and other LMICs, and to share lessons learned during the project implementation.

## Methods

‘The **C**entre for C**a**rdio-Metabolic-**R**isk-**R**eduction in **S**outh-Asia’ (CARRS) studies conduct community-based longitudinal health surveys in three cities (Delhi and Chennai, India and Karachi, Pakistan) comprising 28,000 participants across two urban representative cohorts (CARRS-1 and CARRS-2 initiated in 2010 and 2014, respectively). The CARRS data and samples in India are collected and stored by the Public Health Foundation of India (PHFI) and the Madras Diabetes Research Foundation (MDRF), Chennai, India, in collaboration with the All India Institute of Medical Sciences (AIIMS), India and Emory University, USA. Comprehensive information on household and socio-demographic characteristics, lifestyle factors, medical, family and reproductive histories and quality-of-life was obtained at baseline via interview from men and non-pregnant women aged 20–69 years and residing in the study areas. A random sub-set of New Delhi participants (n = 2045) of CARRS-2 were enrolled in the Oral Health Study between 2014 and 2016 after they filled out a World Health Organization’s Oral Health Assessment questionnaire and underwent extra- and intra-oral clinical examination by qualified dentists assisted by trained field personnel. The purpose of the oral clinical examination was to assess oral hygiene and dental condition, identify mucosal potentially malignant disorders and other lesions and to perform gingival and periodontal health assessment [22]. Dentists attended systematic training and calibration workshops for interpretation of oral indices including Decayed, Missing, Filled Tooth (DMFT), Community Periodontal Index (CPI), and loss-of-attachment (LOA). Gingival and periodontal health was assessed using CPI and LOA on six index teeth. Prevalence of periodontitis was measured according to the U.S. Centres for Disease Control and Prevention and the American Academy of Periodontology [23]. Oral Leukoplakia was clinically diagnosed as a predominantly white lesion of the oral mucosa that cannot be characterized as any other definable lesion by eliminating other mucosal disorders by examination (visual exam and palpation) and history as defined by the International Agency for Research on Cancer by experts at the workshop coordinated by the World Health Organization Collaborating Centre for Oral Cancer and Precancer in the United Kingdom [24].

### Study participants

**Fig 1:**
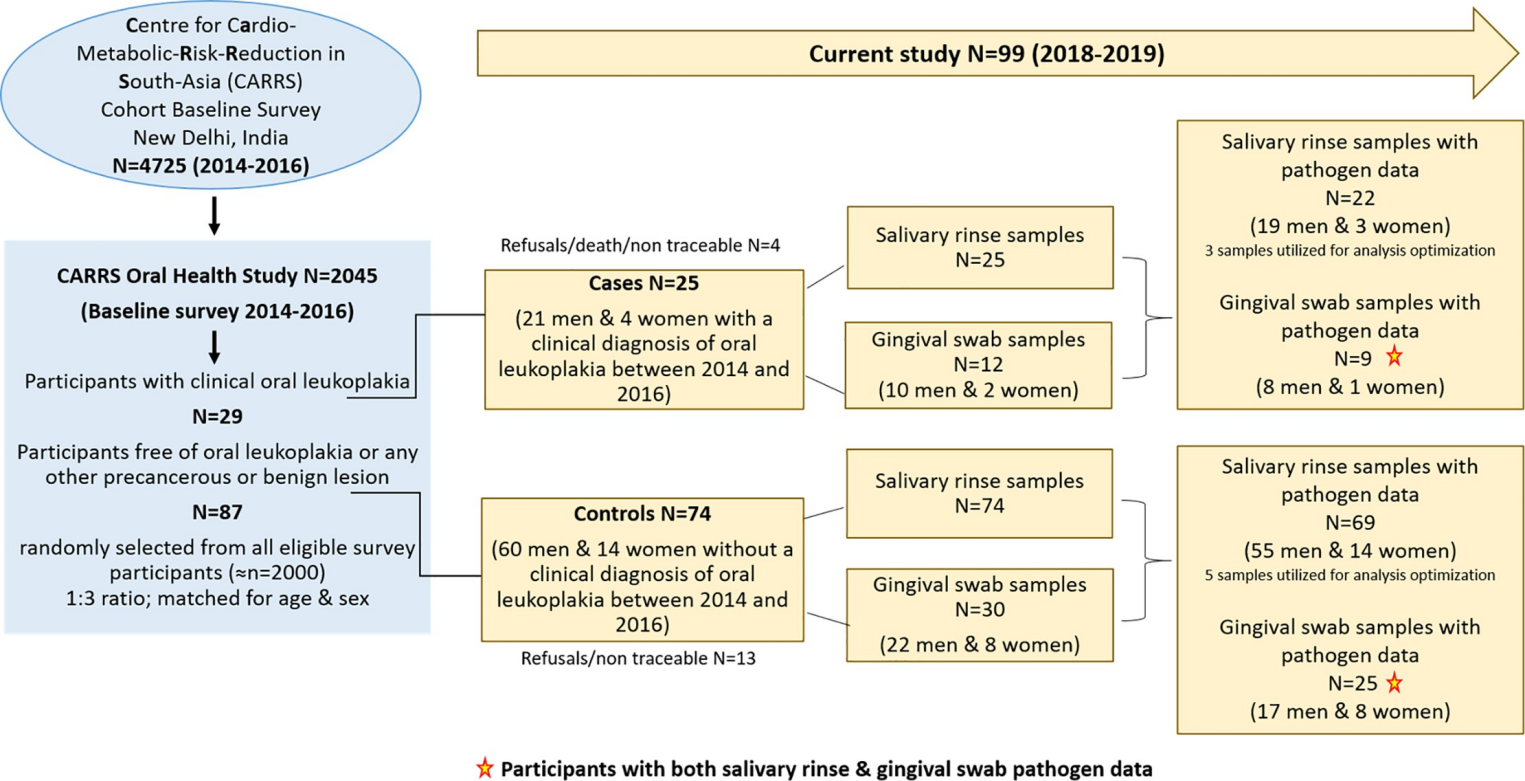
Details of study participants, salivary rinse and gingival swab samples and pathogen data.

Oral Health Study participants selected for the current analyses included 25 persons diagnosed with oral leukoplakia between 2014 and 2016 (cases) and 74 randomly selected cohort members without evidence of oral leukoplakia during the same period (controls). Controls were matched to cases on age and sex. These individuals were re-contacted between November, 2018 and April, 2019 to collect 30 ml of salivary rinse samples and to administer a brief questionnaire on demographic information, tobacco and betel quid use as well as oral health and treatment status. Additionally, gingival swab samples were collected from keratinized gingival surface in a random sub-set of 12 cases and 30 controls (**Fig 1**). Participants refrained from drinking (other than water), eating, chewing, tooth brushing and tobacco use at least 2 hours prior to sampling. The participants did not take alcohol up to 12 hours and any oral antibiotics up to 3 months before sample collection and did not suffer from any acute oral condition or systemic illness at the time of sample collection. Standard protocol under aseptic conditions was followed during sample collection.

### Salivary rinse

Before sample collection, participants cleansed their mouth with water to remove any food residue, and the sample was collected after 10 minutes. Participants were then instructed to rinse the mouth with 30 ml of sterile normal saline for 30 seconds, gargle for 15 seconds, and spit the solution in the sterile 50 ml falcon tube at the end of the sample collection cycle [12,25].

### Gingival swab

Commercially available sterile oral swab (Histogenetics^Rx^) was used to collect swab samples by applying light pressure with the foam tip on the surface of masticatory mucosa between crest gingiva and the mucogingival border [26,27], covering the region of three teeth at once and swiping side to side and up to down (or down to up in lower jaw) for about 10 seconds in total. Entire keratinized gingival region of upper and lower jaw was swiped and the swabs (2 per participant) were immediately pressed against the wall of a 15 ml sterile falcon tube with 2 ml of 1X phosphate buffered saline (PBS; HiMedia^Rx^) and then transferred to the tube. Swab was applied before salivary rinse collection. A gap of 5 minutes was maintained between collection of swab and salivary rinse samples.

All samples were transported at 4°C to the PHFI laboratory within 4 hours of collection. Salivary rinse samples were immediately processed by centrifugation at 4000 x g for 20 minutes at 4°C to separate the cellular pellet from cell-free salivary supernatant. Supernatant was distributed equally in three 15 ml labelled falcon tubes. Cellular pellets were re-suspended in sterile 0.5–1 ml of 1 X PBS (pH 7.2; HiMedia^Rx^) by gently tapping the tube and briefly vortexing it. Care was taken to prevent the pellet fragments from sticking to the tube walls. The samples were then aliquoted evenly into 2 ml Eppendorf tubes. Swab samples were also immediately processed by centrifugation at 4000 x g for 20 minutes at 4°C. Swabs were extracted and stored in 1X TE buffer (10Mm Tris-HCl and 1Mm EDTA-Na_2_, pH 8.0; molecular grade chemicals). Cellular pellets were re-suspended in 1 X PBS present in collection tubes and aliquoted evenly into 2 ml Eppendorf tubes. All samples were stored at -80˚C until further processing. Frozen aliquotes containing 300 μL of suspended cellular pellets were transferred to Molecular Diagnostics Research laboratory for qPCR analysis in storage boxes in dry ice. The remaining samples and supernatant were stored at −80°C for long-term storage at the PHFI bio-repository.

### DNA isolation and quantification

Frozen cellular suspension was thawed at room temperature. Total microbial DNA was extracted from samples using Qiagen^Rx^ (Hilden, Germany) DNA mini kit as per manufacturer’s instruction. 6 μl mutanolysin (25 KU/ml, Sigma-Aldrich) was added to 300 μl aliquot of cells and the mixture was incubated for 30 min at 37°C. After this, 50 μl Proteinase K (20 mg/ml) and 500 μl AL buffer (Qiagen^Rx^, Hilden, Germany) were added and samples were incubated for 30 min at 56°C. 500 μl of ethanol was added and DNA purified by using the columns provided in the kit. The isolated DNA was quantified using fluorescence based Qubit4 (ThermoFisher, Waltham, MA, USA) system and stored at -80˚C till further processing. Thawed sample (2 μl) was used directly into the qPCR reaction. A synthetic control gene block was used as positive control and standard for estimation of bacterial load in the samples.

### qPCR

Salivary rinse and swab levels of *P*. *gingivalis*, *F*. *nucleatum* and *P*. *intermedia* were measured by qPCR. The primers for detection were designed using online tools of National Centre for Biotechnology Information (NCBI) and verified in-silico for melting temperature and secondary structures using Integrated DNA Technology tools (IDT: Coralville, Iowa, USA). The sequences of the primers and probes were derived from specific genes of bacterial pathogens to be able to detect almost all the strains of the species. Mostly, 16S rRNA genes were used for selection of specific regions, which are conserved in most of the strains of the same species making sure that primers were not able to bind non-specifically at any other strain or species of bacteria. For primers and probe designing, genomic sequences following reference strain were used, *P*. *gingivalis* ATCC 33277; *F*. *nucleatum* ATCC 25586; and *P*. *intermedia* ATCC 25611.

We designed the test to be used as multiplex-PCR for the detection of *P*. *gingivalis* and *F*. *nucleatum* together. The synthetic controls for the three pathogens were synthesized from IDT in the form of a gBlock and the primers and probes were synthesized from Sigma-Aldrich^Rx^ (St. Louis, MO, USA). The probes for *P*. *gingivalis* were labeled with fluorescent dye 6-FAM at 5’-end and at the same end probes for *F*. *nucleatum* were labeled with JOE. We quantified *P*. *intermedia* on SYBR Green in a separate set of experiments using the same amount of sample and dilutions of synthetic control as standards. The concentration of control was verified on Qubit4 before preparing serial dilutions in the elution buffer (10mM Tris-HCl pH8.0) to be used as standards.

The qPCR was setup using ThermoFisher Fast Advanced TaqMan-MasterMix (2x) in 20 ul PCR reactions performed on StepOnePlus (ThermoFisher) system for *P*. *gingivalis* and *F*. *nucleatum* while SYBR Green chemistry with 2x PowerUp SYBR Green (ThermoFisher) on the same system was used for *P*. *intermedia*. The sequences of primers and probes and the details of qPCR conditions are presented in **S1 Table**.

The bacterial loads were calculated as per the standard curve generated by the dilutions of standard control diluted serially with a factor of ten from 10^7^ to 10^2^ copies/μl. The amount of bacterial load were estimated for 2μl volume of the sample and then calculated as per nanogram concentration of the total DNA obtained from the sample. Each sample was run in duplicates and the mean critical threshold cycle value was considered for estimation of bacterial load using standard curve method. The critical threshold cycle (Ct) is defined as the first cycle in which fluorescence is detectable above the background, and is inversely proportional to the logarithm of the initial number of template molecules. A standard curve of the Ct values obtained from serial dilutions of control standard on y-axis and log concentration of known bacterial copy numbers on x-axis were plotted to derive a linear regression equation [y = mx+b or Ct = m (log quantity)+b] and the copies of bacterial loads were estimated using Ct value of each sample of DNA obtained from salivary rinse and gingival swab. The detection of pathogen is defined by the positive mean critical threshold cycle (Ct) value; and the quantification of pathogen is defined by positively calculated genome copies/ng of DNA for the available DNA concentration of the sample.

### Statistical analysis

All data were presented as means (standard deviations), medians (interquartile ranges) and counts (proportions). The levels of oral pathogens were expressed as genome copies/ng of DNA. The primary outcome (oral leukoplakia) was binary (yes/no). The other risk factors were presented as follows: age (in years), sex (male/female), SES (tertiles), tobacco use (yes/no), alcohol consumption (yes/no), diet type (vegetarian/non-vegetarian) and periodontal disease (yes/no). The differences in means, proportions and medians between cases and controls were tested using t-tests, chi-square, Wilcoxon rank-sum or Kruskal-Wallis ANOVA tests, depending on the variable of interest. Pearson correlation coefficient was calculated for each pair of pathogens in cases and controls. All microbial measures were examined across sociodemographic characteristics, lifestyle factors and periodontal disease status among controls. Sensitivity analyses were performed separately after excluding 22 participants (5 cases and 17 controls) with any oral treatment history in the past year or any medical treatment for leukoplakia since the baseline survey. All analyses were conducted using STATA statistical package version SE15 (Stata-Corp.2015.Stata Statistical Software: Release 15. StataCorp LP).

### Ethics statement

All study participants provided informed consent. Information sheets in local language were given to the participants, and their signatures were obtained in the consent forms. Ethical approval for the study was obtained from the Centre for Chronic Disease Control Institutional ethics committee (Number: CCDC-IEC_08_2017).

## Results

### Participant characteristics

**Table 1** summarizes the baseline characteristics of 25 participants with a history of clinical oral leukoplakia (cases) and 74 persons without a history of clinical oral leukoplakia (controls). Compared to controls, cases were significantly (p<0.05) more likely to be of low socioeconomic status, use tobacco and adhere to a non-vegetarian diet. The two groups were similar with respect to age, gender, employment status, alcohol use and prevalence of clinically diagnosed periodontal disease.

**Table 1:** Sociodemographics, life-style, medical and oral health characteristics of study participants with and without a clinical diagnosis of oral leukoplakia between 2014 and 2016 (N = 99).

Characteristics Number (%)	Participants with clinical oral leukoplakia	Participants without clinical oral leukoplakia	p-value*
	(Cases N = 25)	(Controls N = 74)	
Age, mean (±SD)	45.7 (9.8)	44.6 (8.6)	0.60
Number of men (%)	21 (84%)	60 (81%)	0.74
Education			
No formal education/illiterate	6 (24%)	9 (12%)	0.15
School/college Education	19 (76%)	65 (88%)	
Occupation			
Unemployed	9 (38%)	18 (25%)	0.25
Employed	15 (63%)	53 (75%)	
Socioeconomic score (median, IQR)	-1.6 (-2.9, 0.8)	0.3 (-1.0, 2.1)	0.017
Tobacco use in any form			
Never	4 (16%)	48 (65%)	<0.001
Ever	21 (84%)	26 (35%)	
Alcohol use			
Never	13 (52%)	43 (58%)	0.59
Ever	12 (48%)	31 (42%)	
Diet			
Non-vegetarian	18 (72%)	35 (47%)	0.032
Vegetarian	7 (28%)	39 (53%)	
Clinically diagnosed periodontal disease			
No	12 (48%)	49 (66%)	0.11
Yes	13 (52%)	25 (34%)	

*t-test, Chi-Square test and Wilcoxon Rank-Sum test for differences in mean, proportion and median respectively.

### Distribution and levels of selected oral bacterial pathogens in salivary rinse

The analyses of bacterial pathogens included 22 cases and 69 controls who provided salivary rinse samples. **Table 2** presents three parameters for each oral bacterial pathogen measured in salivary rinse samples: the proportion of detection, the proportion of quantified samples, and the levels (genome copies/ng of DNA) of the pathogen. In the total study population, the proportion of detection of oral bacterial pathogens in salivary rinse samples was 100% for *P*. *gingivalis*, 99% for *F*. *nucleatum* and 55% for *P*. *intermedia;* and the proportions with quantifiable levels of oral bacterial pathogens for *P*. *gingivalis*, *F*. *nucleatum* and *P*. *intermedia* were 97%, 84% and 53%, respectively. About a half of the participants had all three pathogens detected and quantified.

**Table 2:** Distribution of *P*. *gingivalis (Pg)*, *F*. *nucleatum (Fn)* and *P*. *intermedia (Pi)* in salivary rinse samples of participants with and without a clinical diagnosis of oral leukoplakia between 2014 and 2016 (N = 91).

Number (%)	Cases N = 22	Controls N = 69	p-value*	Total participants N = 91
*Pg* detected**	22 (100%)	69 (100%)	-	91 (100%)
*Pg* quantified	21 (95%)	67 (97%)	0.71	88 (97%)
*Pg* copies/ng of DNA median (IQR)	1.24x10^4^ (3.29x10^3^, 2.51x10^4^)	9.82x10^3^ (4.81x10^3^, 2.52x10^4^)	0.97	1.00x10^4^ (4.54x10^3,^ 2.51x10^4^)
*Fn* detected**	22 (100%)	68 (99%)	0.57	90 (99%)
*Fn* DNA quantified	19 (86%)	57 (83%)	0.68	76 (84%)
*Fn* copies/ng of DNA median (IQR)	1.52 x10^4^ (5.33x10^3^, 2.39x10^4^)	1.55x x10^4^ (7.94 x10^3^, 2.67 x10^4^)	0.59	1.53x10^4^ (7.34x10^3^, 2.65x10^4^)
*Pi* detected***	15 (68%)	35 (51%)	0.15	50 (55%)
*Pi* quantified	14 (64%)	34 (49%)	0.24	48 (53%)
*Pi* copies/ng of DNA median (IQR)	1.68x10^4^ (1.29x10^4^, 2.41x10^4^)	2.51x10^4^ (1.30x10^4^, 5.13x10^4^)	0.39	2.09x10^4^ (1.30x10^4^, 4.86x10^4^)
Any one pathogen detected	22 (100%)	69 (100%)	-	91 (100%)
Any one pathogen quantified	21 (95%)	68 (99%)	0.38	89 (98%)
All three pathogens detected	15 (68%)	34 (49%)	0.12	49 (54%)
All three pathogens quantified	12 (55%)	29 (42%)	0.30	41 (45%)
Total pathogen copies/ng of DNA median (IQR)	3.59x10^4^ (1.80 x10^4^, 5.97x10^4^)	3.03 x10^4^ (1.70x10^4^, 8.67x10^4^)	0.75	3.11x10^4^ (1.77x10^4^, 7.84x10^4^)

*Chi-Square test and Wilcoxon Rank-Sum test for differences in proportion and median respectively.

**Taqman assay

***Sybr Green assay.

The proportion of detection and quantification as well as the levels of *P*. *gingivalis*, *F*. *nucleatum*, *P*. *intermedia* and total pathogens did not vary between cases and controls. We categorized the participants into tertiles based on the quantified levels for each pathogen. The proportion of cases and controls for any of the three pathogens did not differ by categories. However, among participants with high levels of pathogens (i.e., in second and third tertiles), *P*. *gingivalis* levels tended to be higher in cases than in controls (**S1 & S2 Figs**).

**Fig 2:**
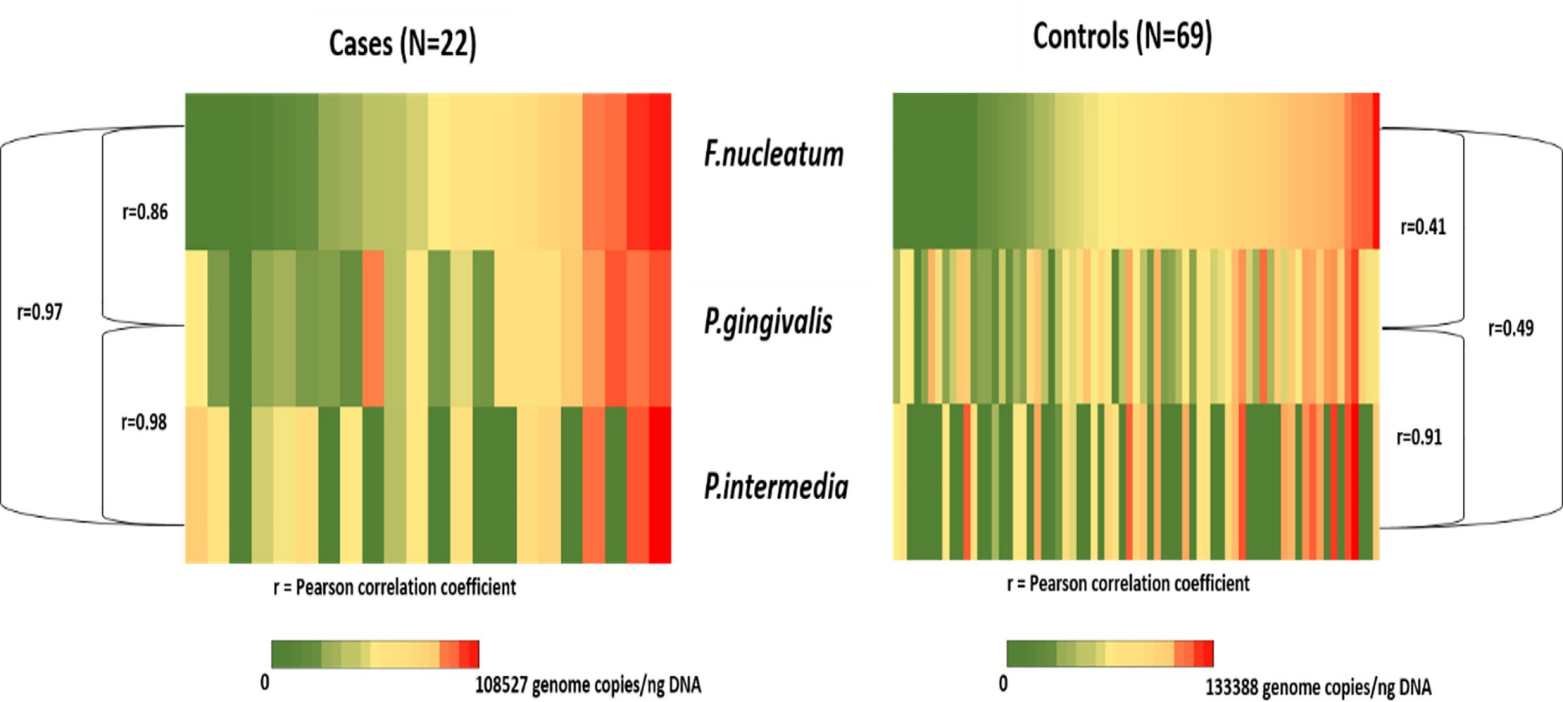
Heat-map showing distribution and correlations of *P*.*gingivalis*, *F*.*nucleatum* and *P*.*intermedia* genome copies in salivary rinse samples of the study participants (N = 91).

The levels of total and individual pathogens in salivary rinse samples of cases ranged between 7.94×10^3^ and 2.83×10^5^ copies/ng for total pathogens; 1.32×10^3^ and 7.60×10^4^ copies/ng for *P*. *gingivalis*; 3.34×10^2^ and 9.84×10^4^ copies/ng for *F*. *nucleatum*; and 7.10×10^3^ and 1.08×10^5^ copies/ng for *P*. *intermedia*. The levels of individual pathogens were highly correlated with each other (**Fig 2**) with Pearson correlation coefficients in the 0.86–0.98 range (all p-values <0.0001).

The levels of total and individual pathogens in salivary rinse samples of controls ranged between 3.04×10^3^ and 3.14×10^5^ copies/ng for total pathogens; 3.17×10^2^ and 1.01×10^5^ copies/ng for *P*. *gingivalis*; 1.17×10^3^ and 1.26×10^5^ copies/ng for *F*. *nucleatum*; and 1.65×10^3^ and 1.33×10^5^ copies/ng for *P*. *intermedia*. These levels also correlated but to a lesser extent than among cases; with Pearson coefficients of 0.41 (p = 0.0017) for *P*. *gingivalis-F*. *nucleatum*, 0.91 (p<0.0001) for *P*. *gingivalis-P*. *intermedia*, and 0.49 (p = 0.0067) for *P*. *intermedia-F*. *nucleatum* (**Fig 2**).

The distributions and levels of *P*. *gingivalis*, *F*. *nucleatum* and *P*. *intermedia* among control participants did not differ by sex, tobacco or alcohol use, dietary pattern or presence of clinical periodontal disease (**S1–S7 Tables**). The total pathogen levels were three-times higher among participants with low socio-economic status compared to the rest of the study population (**S3 Table**). The proportion of detection of all three pathogens was 1.5 fold higher among men than women (**S2 Table**); however, this difference was not statistically significant.

### Comparison of salivary rinse and gingival swab samples for the distribution and levels of selected oral bacterial pathogens

**Table 3** compares the three parameters under study for each oral pathogen in salivary rinse and gingival swab samples. These analyses were performed using data on 34 participants (9 cases and 25 controls) with both types of samples available. The proportion of pathogen detection did not differ between the two types of samples except for *P*. *intermedia* and all three pathogens combined; however, the proportion of quantifiable pathogens was better (p<0.05) in salivary rinses compared to gingival swabs. Depending on the pathogen, this proportion ranged from 44% to 94% in salivary rinse samples and from 3% to 29% in gingival swabs. The mean concentration of bacterial pathogen DNA was significantly higher (p<0.001) in salivary rinse samples (range: 0.2–6 ng/μl) than in gingival swabs (range: 0.05–0.5 ng/μl).

**Table 3:** Comparison of distribution of *P*. *gingivalis (Pg)*, *F*. *nucleatum (Fn)*, *P*. *intermedia (Pi)* in salivary rinse versus gingival swab samples (N = 34 participants; 9 cases and 25 controls).

Number (%)	Salivary rinse N = 34	Gingival swab N = 34	p-value*
*Pg* detected**	34 (100%)	33 (97%)	0.31
*Pg* quantified	32 (94%)	10 (29%)	<0.001
*Pg* copies/ng of DNA, median (IQR)	1.10x10^4^ (5.7x10^3^, 2.33x10^4^)	4.31x10^4^ (1.05x10^4^, 1.28x10^5^)	0.010
*Fn* detected**	34 (100%)	32 (94%)	0.15
*Fn* DNA quantified	29 (85%)	4 (12%)	<0.001
*Fn* copies/ng of DNA, median (IQR)	1.72x10^4^ (8.85x10^3^, 2.99x10^4^)	4.79x10^4^ (1.34x10^4^, 8.27x10^4^)	0.29
*Pi* detected***	17 (50%)	9 (26%)	0.046
*Pi* quantified	15 (44%)	1 (3%)	<0.001
*Pi* copies/ng of DNA, median (IQR)	1.79x10^4^ (1.38x10^4^, 4.35x104)	1.37x10^5^ (n = 1)	-
Any one pathogen detected	34 (100%)	34 (100%)	-
Any one pathogen quantified	32 (94%)	10 (29%)	<0.001
All three pathogens detected	17 (50%)	8 (24%)	0.024
All three pathogens quantified	14 (41%)	Nil	<0.001
Total pathogen copies/ng of DNA median (IQR)	3.58x10^4^ (1.94x10^4^, 7.80x10^4^)	6.31x10^4^ (2.16x10^4^, 1.57x10^5^)	0.23
DNA concentration ng/μl, mean (SD)	1.8 (1.7)	0.2 (0.1)	<0.001

*Chi-Square test and Wilcoxon Rank-Sum test for differences in proportion and median respectively.

**Taqman assay

***Sybr Green assay.

When we evaluated the findings excluding participants who had undergone any dental treatment in the past one year or had any medical treatment for leukoplakia anytime since the baseline survey, all our results were consistent with those observed in the full study (*data not shown*).

## Discussion

This study allowed us to test the feasibility of the study design, sampling strategy and data collection procedures including sample storage, processing and analyses, to assess the role of oral microbiome for oral cancer risk in the general population of LMICs. In this study, we confirmed previously reported associations between clinical oral leukoplakia and established risk factors such as low socioeconomic status and tobacco use, as well as possibly a non-vegetarian diet [17]. Although the distributions or the levels of *P*. *gingivalis*, *F*. *nucleatum* and *P*. *intermedia* were not associated with previously diagnosed clinical oral leukoplakia, stronger correlations between the levels for any two pathogens were seen among leukoplakia cases compared to leukoplakia-free controls. These findings were consistent or stronger after excluding participants who self-reported having received any oral treatment.

Several plausible cancer-initiating and promoting mechanisms of these bacterial pathogens, based on animal models, exist. These mechanisms include direct DNA damage through endotoxins, activation of cell-cycle signalling through toll-like receptors as well as the promotion of local and systemic inflammation [2,14,28]. The effects of these oral pathogens may be independent of other factors or act synergistically with other known carcinogens (e.g., tobacco/alcohol/diet) [4,29]. The ability of these pathogens, particularly *P*. *gingivalis*, to evade host immune mechanisms enables them to survive in systemic circulation and reach distant organ sites [2]. Due to these properties, emerging evidence shows positive associations of oral bacterial pathogens not only with oral potentially malignant lesions [5,8] and oral or orodigestive cancers, [30–33] but also with distant cancer sites [1,34,35]. Recent meta-analysis on the effect of periodontal bacteria infection on incidence and prognosis of cancer confirmed that *P*.*gingivalis* and *P*.*intermedia* infection was associated with high incidence of cancer and *P*.*gingivalis* and *F*.*nucleatum* infection was associated with poor prognosis [36].

The absence of observable differences in distribution and levels for individual or total pathogens between cases and controls in our study indicates that these pathogens might not play an important role in a population with a high prevalence of established risk factors such as tobacco use. The observed results might have also been affected by systematic error, such as selection bias, uncontrolled confounding or insufficient statistical power. However, the preliminary findings such as ours are necessary to assess the feasibility for designing and implementing full-scale population based research.

It is important to consider that the composition of pathogens may be more informative than the distributions of individual microorganisms. In the Buffalo Osteoporosis and Periodontal Disease (OsteoPerio) cohort study of postmenopausal women, no associations were found for individual pathogens, but, the presence of pathogen-complex (*F*. *nucleatum*, *P*. *intermedia*, and *C*. *rectus*) appeared to be positively associated with total cancer risk, highlighting the possibility of synergistic interactions among several pathogens [37]. These findings are in broad agreement with the results of our analyses showing stronger inter-correlations of pathogen levels in leukoplakia cases than in controls. High-throughput sequencing data from epidemiological studies for the associations of this pathogen-complex with oral or head and neck cancers and leukoplakia are currently mixed but still emerging [4–11,38,39].

We also found that in two-thirds of our participants with relatively high levels of pathogens, the levels of *P*. *gingivalis* tended to be higher in cases than in controls. This finding also merits further exploration in a larger study. Cohort studies in pancreatic and orodigestive cancers have found increased risk for incidence and mortality with high levels of *P*. *gingivalis* antibodies in serum, [1] and similarly high levels of *F*. *nucleatum* in saliva appear to be associated with colon cancer risk [40].

Our study participants, in general had a high prevalence for *P*. *gingivalis* (>90%) and *F*. *nucleatum* (>80%) as well as for *P*. *intermedia* (>50%), with no differences between cases and controls either for individual or total pathogens. This is in contrast to a recent sequencing study that found significant difference in the proportions and relative abundance of these pathogens between cases of oral leukoplakia or cancer and controls (88% vs. 33%) (5). Another recent study conducted in Turkey, reported a high prevalence for *P*. *gingivalis* (>75%) and *F*. *nucleatum* (>95%) both in colon cancer cases and controls [40]. By contrast, an earlier study of pancreatic cancer reported case-control differences for both *P*. *gingivalis* (35.5% vs. 25.9%) and *P*. *intermedia* (22.7% vs. 18.9%) [41]. The high prevalence of pathogens in our study may reflect the widespread periodontal disease and poor oral health in the general population of India [22,42]. This may be of importance, because there is evidence that the effect of bacterial pathogens on oral cancer risk may differ in individuals with and without periodontal disease [6,30,43,44]. Further, the carcinogenic potential of these pathogens may be more evident among non-users of tobacco or alcohol [5,45,46]. The limited scope of our study prevented a more in-depth analysis of the individual and joint effects of microbial, lifestyle and oral health related factors.

The sensitivity of analytic methods may also influence the detection rate [47,48]. The probes in our study were designed to detect low levels (up to 100 copies/μl of sample) of all strains of the selected pathogens. It is important to point out that the methods for detecting *P*. *intermedia* were different from those used to detect *P*. *gingivalis*, and *F*. *nucleatum*. *P*. *intermedia* detection was based on the SYBR Green method, whereas the other two pathogens were examined using a more sensitive Taqman assay. Nevertheless, both these assays are validated to perform equally well under standardized conditions [48,49]. While SYBR Green assay could have influenced the overall low detection and quantification of *P*.*intermedia*, it is unlikely to have produced differential measurement error in cases and controls. Thus, our findings, in line with existing literature, indicate that the composition and the levels of pathogens may be more important than detection rates [13].

From the study implementation and dissemination perspective, we found that salivary rinse might be better suited for quantitative microbial qPCR analysis compared to gingival swabs. This was primarily due to poor bacterial DNA quality from gingival swab samples. The current literature offers few examples of such comparisons. Smola et al in 2003 [26] observed that gingival swabs were an easy and reliable method for PCR detection of oral pathogens; however, quantitative analysis in that study was not done. Recently, in another study, authors found that gingival swabs were comparable to salivary rinse samples in terms of viral DNA detection rates although quantification levels were higher in salivary rinse [27]. Thus, methodology in population-based studies might depend on the type of analysis (qualitative versus quantitative) as well as the pathogen of interest (e.g. viral versus bacterial). Interestingly, swab samples yield poor quality human DNA compared to salivary rinse samples [50,51]. Novel DNA extraction-free qPCR methods for swab analyses seem to offer a good alternative [52] that should be explored in population based microbiome studies.

Additional noteworthy findings from our study include higher levels of total pathogens among participants of lower socioeconomic status and the higher prevalence of all three pathogens detected among men. The results related to the socioeconomic differences appear to be consistent with high-throughput analyses in Western populations, wherein, these pathogens were correlated with socioeconomic status even after adjusting for smoking, oral health status and diet [53]. The gender differences in pathogens appear to be attributable primarily to *P*. *intermedia*. In general, the levels of *P*. *intermedia* were higher than the levels of the other two pathogens in our study.

Perhaps the most important limitation of our study is the time lag (range: 2–4.5 years) between the leukoplakia diagnosis and the collection of salivary rinse and gingival swab samples. It is possible that dental treatment and/or lifestyle modification changed the microbiome prevalence and levels. However, oral microbiota exhibit the feature of ‘stationary dynamics’ and the composition fluctuates around a stable mean and remains relatively consistent within a 10 year period [54–56]. This makes oral pathogens promising biomarkers for early cancer detection, as well as monitoring of disease progression and prognosis [57–59]. Although this property has not yet been studied in relation to cancer or precancerous lesions at population level, cohorts with potential to study early changes in carcinogenesis can be a good resource of data for temporal analysis. The time-lag could also have influenced leukoplakia status at the time of sample collection leading to misclassification of controls. During control selection, we carefully excluded participants with any oral lesion at baseline as recorded by the dentists (including benign or tobacco associated lesions). During follow-up visits, the trained field health workers ruled out any oral cavity lesions before collecting saliva samples. Control participants were ineligible if they had any lesion. A detailed history regarding dental visits and treatment as well as current tobacco use was obtained for all cases and controls. Our data indicate that about a fifth of the study participants had undergone any dental treatment in the past one year or had treatment for leukoplakia at any time since the baseline. Sensitivity analysis after excluding these participants did not change the findings, although in some instances the difference between cases and control became more pronounced. We also collected information on tobacco use, the single most important risk factor for oral cancer. A little over 10% of participants who classified never/ever tobacco users at baseline changed their classification based on current use of tobacco products. A re-analysis of prevalence and levels of pathogens based on current tobacco and betel quid use status produced results that were similar to those obtained using baseline data. Lack of pathological confirmation of leukoplakia is another limitation, however, clinical diagnosis is an independent confirmatory diagnosis for population-based studies [60]. The study participants completed a WHO Oral Health Assessment questionnaire which was previously used in population-based studies [61] and underwent intra-oral clinical examination by qualified dentists who were assisted by trained field personnel. Oral leukoplakia was clinically diagnosed as a predominantly non-scrapable white lesion of the oral mucosa that cannot be characterized as any other definable lesion by eliminating other mucosal disorders by examination from a single visit, including the clinical presentation of the lesion [62]. Factors such as age, sex, lifestyle factors, as well as history of previous dental visits and treatments and oral hygiene practices were taken into consideration. Medical records of participants, who had a previous dental visit and/or a leukoplakia diagnosis from a dentist or physician, were assessed. Common conditions such as oral candidiasis and any source of irritation were excluded on oral examination. Such comprehensive unaided clinical examination by dental surgeons has been found to have accuracy (98%) and reliability (87%) as comparable to clinical specialists [63]. Pathology confirmation of cases at a population-level is generally not feasible and studies this type are usually conducted without biopsy [60,62].

## Conclusions

In summary, the current study confirms the feasibility of evaluating characteristics of microbial flora in population-based studies conducted in low resource settings. Importantly, this study was nested within one of the largest existing South Asian cohort with a bio-repository that allows evaluating other potentially modifiable and targetable risk factors for oral neoplasia. We further showed that salivary rinse might serve as a better medium for quantitative microbial analysis compared to gingival swabs. The experience gained during the development and implementation of this pilot study will inform future research in India and in other countries that experience a high burden of oral and orodigestive cancers.

## Supporting information

S1 Fig Comparison of median levels of bacterial pathogens between cases and controls among participants with high levels of pathogens (i.e., in the second tertile). (DOCX)

S2 Fig Comparison of median levels of bacterial pathogens between cases and controls among participants with high levels of pathogens (i.e., in the third tertile). (DOCX)

S1 Table Sequences of primers and probes of oral bacterial pathogens and qPCR Conditions. (DOCX)

S2 Table Distribution of *P*.*gingivalis (Pg)*, *F*.*nucleatum (Fn)* and *P*.*intermedia (Pi)* in salivary rinse samples among participants without a clinical diagnosis of oral leukoplakia between 2014 and 2016 by sex (N = 69). (DOCX)

S3 Table Distribution of *P*.*gingivalis (Pg)*, *F*.*nucleatum (Fn)* and *P*.*intermedia (Pi)* in salivary rinse samples among participants without a clinical diagnosis of oral leukoplakia between 2014 and 2016 by socioeconomic status (N = 69). (DOCX)

S4 Table Distribution of *P*.*gingivalis (Pg)*, *F*.*nucleatum (Fn)* and *P*.*intermedia (Pi)* in salivary rinse samples among participants without a clinical diagnosis of oral leukoplakia between 2014 and 2016 by tobacco use status in any form (N = 69). (DOCX)

S5 Table Distribution of *P*.*gingivalis (Pg)*, *F*.*nucleatum (Fn)* and *P*.*intermedia (Pi)* in salivary rinse samples among participants without a clinical diagnosis of oral leukoplakia between 2014 and 2016 by alcohol use status (N = 69). (DOCX)

S6 Table Distribution of *P*.*gingivalis (Pg)*, *F*.*nucleatum (Fn)* and *P*.*intermedia (Pi)* in salivary rinse samples among participants without a clinical diagnosis of oral leukoplakia between 2014 and 2016 by dietary pattern (N = 69). (DOCX)

S7 Table Distribution of *P*.*gingivalis (Pg)*, *F*.*nucleatum (Fn)* and *P*.*intermedia (Pi)* in salivary rinse samples among participants without a clinical diagnosis of oral leukoplakia between 2014 and 2016 by periodontal disease status (N = 69). (DOCX)
