# Supplementary figures and images for: Feasibility of investigating the association between bacterial pathogens and oral leukoplakia in low and middle income countries: A population-based pilot study in India
Source: PLoS One. 2021 Apr 29;16(4):e0251017. doi: 10.1371/journal.pone.0251017 (PMC8084244; doi:10.1371/journal.pone.0251017)

**
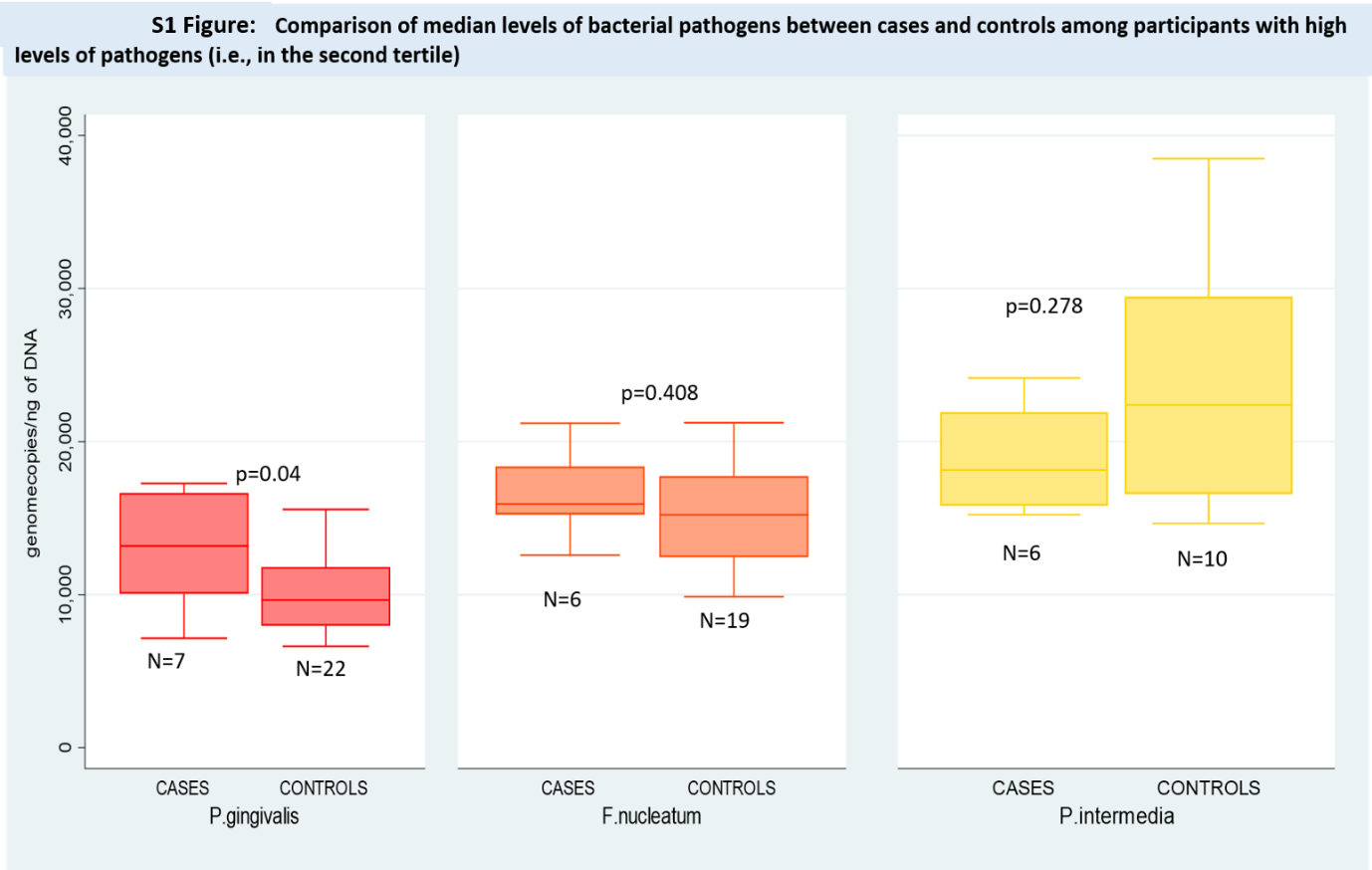
**

Supplement: S1 Fig — (DOCX) [file pone.0251017.s001.docx]

**
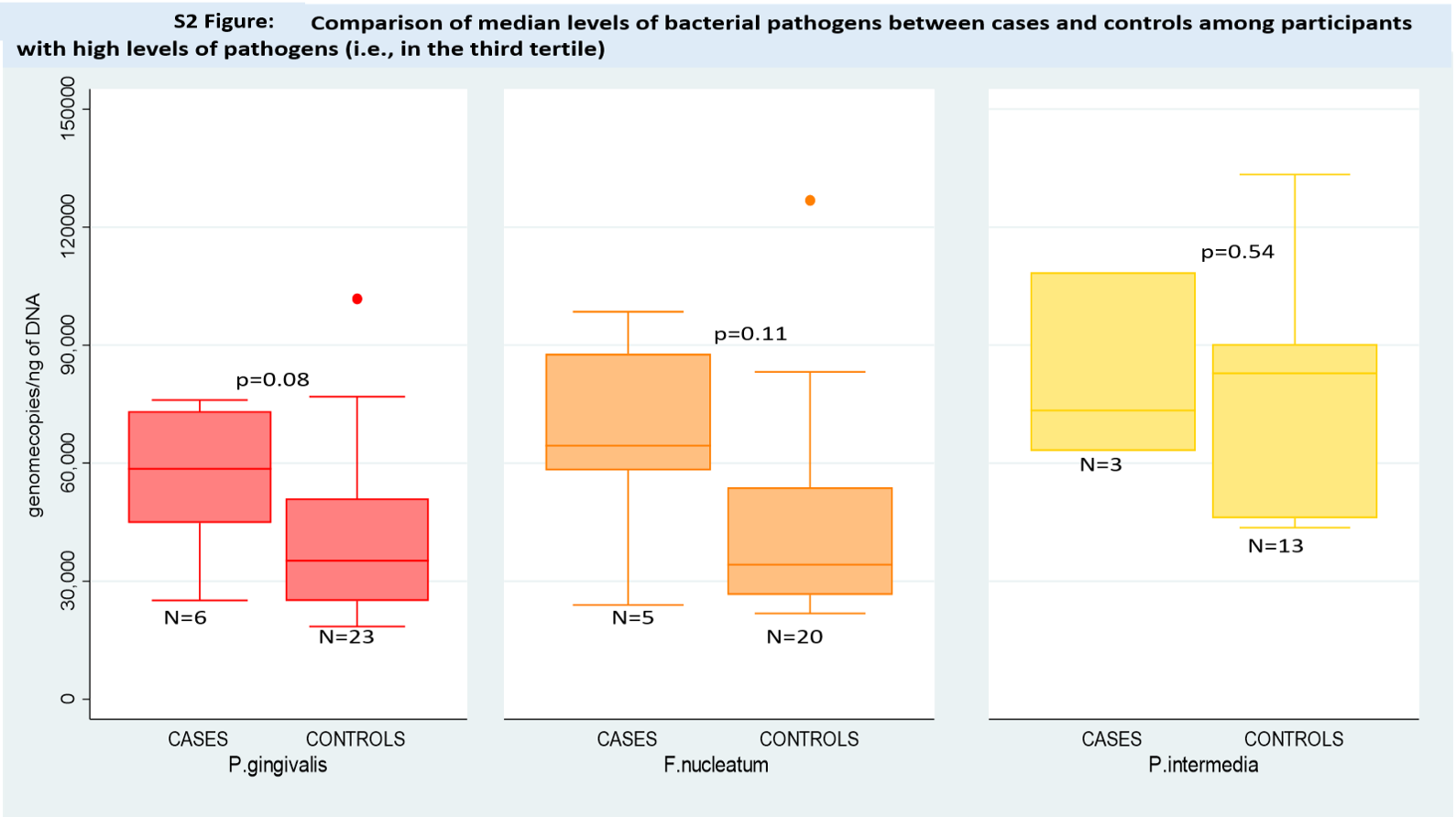
**

Supplement: S2 Fig — (DOCX) [file pone.0251017.s002.docx]
