# Supplementary material for: Feasibility of investigating the association between bacterial pathogens and oral leukoplakia in low and middle income countries: A population-based pilot study in India
Source: PLoS One. 2021 Apr 29;16(4):e0251017. doi: 10.1371/journal.pone.0251017 (PMC8084244; doi:10.1371/journal.pone.0251017)
Supplement: S1 Table — (DOCX) [file pone.0251017.s003.docx]

**S1 Table:**

Sequences of primers and probes of oral bacterial pathogens and qPCR Conditions

| **Pathogen** | **DNA Sequences (5’-3’)** | **Base pair size** | **Melting temperature (°C)** |
| --- | --- | --- | --- |
| *Porphyromonas gingivalis** | Fwd Primer: AGTGTATTGATCACTCAGGAATGTG | 25 | 62.7 |
|  | Rev Primer: CTGATGGTAAGGAGCGATGAAG | 22 | 62.3 |
|  | Probe: **6-FAM**- CTGTCGTCTGTGCAGCCAATCCT | 23 | 68.2 |
| *Fusobacterium nucleatum** | Fwd Primer: GCAAGACAAATTAATACAATAGTAGACCA | 29 | 62.5 |
|  | Rev Primer: CACTCCAAATACTCCAACTGGAAG | 26 | 62.9 |
|  | Probe: **JOE-** TGCAGGTTCAATAACAGCACTTGAAAATGC | 30 | 68.4 |
| *Prevotella intermedia*** | Fwd Primer: GCCAACGAGTACTCCTGATGTAG | 23 | 63.8 |
|  | Rev Primer: AGGTACCGAGAACGACAAGT | 20 | 62.7 |
|  | Chemistry: **SYBR GREEN** | NA | NA |
| ***TaqMan Assay** | **Temperature (°C)** | **Time (mm:ss)** | **Cycles** |
| UNG incubation | 50 | 02:00 | x1 |
| Polymerase activation | 95 | 00:30 | x1 |
| Denaturation | 95 | 00:03 | x40 |
| Primers/Probes annealing and Extension (amplification) | 60 | 00:30 |  |
| ****SYBR Green Assay** | **Temperature (°C)** | **Time (mm:ss)** | **Cycles** |
| UNG incubation | 50 | 02:00 | x1 |
| Polymerase activation | 95 | 02:00 | x1 |
| Denaturation | 95 | 00:15 | x40 |
| Primers/Probes annealing and Extension (amplification) | 60 | 01:00 |  |
