# Supplementary material for: Feasibility of investigating the association between bacterial pathogens and oral leukoplakia in low and middle income countries: A population-based pilot study in India
Source: PLoS One. 2021 Apr 29;16(4):e0251017. doi: 10.1371/journal.pone.0251017 (PMC8084244; doi:10.1371/journal.pone.0251017)
Supplement: S2 Table — (DOCX) [file pone.0251017.s004.docx]

**S2 Table:** Distribution of *P. gingivalis (Pg)*, *F. nucleatum (Fn)* and *P. intermedia (Pi)* in salivary rinse samples among participants without a clinical diagnosis of oral leukoplakia between 2014 and 2016 by sex (N=69)

| Characteristics  Number (%) | Men  N=55 | Women  N=14 | p-value* |
| --- | --- | --- | --- |
| *Pg* detected** | 55 (100%) | 14 (100%) | - |
| *Pg* quantified | 53 (96%) | 14 (100%) | 0.47 |
| *Pg* copies/ng of DNA, median (IQR) | 9.45x10^3^ (4.74x10^3^, 2.49x10^4^) | 1.10x10^4^ (6.62x10^3^, 4.65x10^4^) | 0.45 |
| *Fn* detected** | 54 (98.2%) | 14 (100%) | 0.61 |
| *Fn* quantified | 45 (82%) | 12 (86%) | 0.73 |
| *Fn* copies/ng of DNA, median (IQR) | 1.44x10^4^ (7.38x10^3^, 2.25x10^4^) | 2.25x10^4^ (1.33x10^4^, 3.44x10^4^) | 0.14 |
| *Pi* detected*** | 31 (56%) | 4 (29%) | 0.063 |
| *Pi* quantified | 30 (55%) | 4 (29%) | 0.083 |
| *Pi* copies/ng of DNA, median (IQR) | 2.23x10^4^ (1.30x10^4^, 4.49x10^4^) | 6.47x10^4^ (2.87x10^4^, 9.30x10^4^) | 0.22 |
| Any one pathogen detected | 55 (100%) | 14 (100%) | - |
| Any one pathogen quantified | 54 (98%) | 14 (100%) | 0.61 |
| All three pathogens detected | 30 (55%) | 4 (29%) | 0.083 |
| All three pathogens quantified | 25 (45%) | 4 (29%) | 0.27 |
| Total pathogen copies/ng of DNA  median (IQR) | 2.87x10^4^ (1.67x10^4^, 7.26x10^4^) | 4.09x10^4^ (1.76x10^4^, 1x10^5^) | 0.56 |

*Chi-square test and Wilcoxon Rank-sum test for differences in proportion and median respectively.

**Taqman assay ***Sybr Green assay
