# Supplementary material for: Feasibility of investigating the association between bacterial pathogens and oral leukoplakia in low and middle income countries: A population-based pilot study in India
Source: PLoS One. 2021 Apr 29;16(4):e0251017. doi: 10.1371/journal.pone.0251017 (PMC8084244; doi:10.1371/journal.pone.0251017)
Supplement: S3 Table — (DOCX) [file pone.0251017.s005.docx]

**S3 Table:** Distribution of *P. gingivalis (Pg)*, *F. nucleatum (Fn)* and *P. intermedia (Pi)* in salivary rinse samples among participants without a clinical diagnosis of oral leukoplakia between 2014 and 2016 by socioeconomic status (N=69)

| Characteristics  Number (%) | Tertile-1:Low  N=19 | Tertile-2:Medium  N=22 | Tertile-3:High  N=28 | p-value* |
| --- | --- | --- | --- | --- |
| *Pg* detected** | 19 (100%) | 22 (100%) | 28 (100%) | - |
| *Pg* quantified | 18 (95%) | 22 (100%) | 27 (96%) | 0.58 |
| *Pg* copies/ng of DNA, median (IQR) | 1.32X10^4^  (9.03X10^3^, 2.88X10^4^) | 6.94X10^3^  (4.30X10^3^, 2.41X10^4^) | 1.01X10^4^  (4.35X10^4^, 2.71X10^4^) | 0.23 |
| *Fn* detected** | 19 (100%) | 22 (100%) | 27 (96%) | 0.48 |
| *Fn* quantified | 17 (89%) | 17 (77%) | 23 (82%) | 0.59 |
| *Fn* copies/ng of DNA, median (IQR) | 2.11X10^4^  (9.86X10^3^, 3.37X10^4^) | 1.81X10^4^  (7.94X10^3^, 3.19X10^4^) | 1.37X10^4^  (7.65X10^3^, 2.25X10^4^) | 0.53 |
| *Pi* detected*** | 12 (63%) | 10 (45%) | 13 (46%) | 0.44 |
| *Pi* quantified | 11 (58%) | 10 (45%) | 13 (46%) | 0.68 |
| *Pi* copies/ng of DNA, median (IQR) | 4.35X10^4^  (2.26X10^4^, 8.34X10^4^) | 4.13X10^4^  (1.24X10^4^, 5.13X10^4^) | 1.46X10^4^  (1.24X10^4^, 2.20X10^4^) | 0.15 |
| Any one pathogen detected | 19 (100%) | 22 (100%) | 28 (100%) | - |
| Any one pathogen quantified | 18 (95%) | 22 (100%) | 28 (100%) | 0.26 |
| All three pathogens detected | 12 (63%) | 10 (45%) | 12 (43%) | 0.36 |
| All three pathogens quantified | 10 (53%) | 8 (36%) | 11 (39%) | 0.53 |
| Total pathogen copies/ng of DNA  median (IQR) | 7.55x10^4^  (2.93x10^4^, 1.10x10^5^) | 2.43x10^4^  (1.17x10^4^, 8.35x10^4^) | 2.85x10^4^  (1.76x10^4^, 4.51x10^4^) | 0.038 |

*Chi-square test and Kruskal-Wallis ANOVA test for differences in proportion and median respectively.

**Taqman assay ***Sybr Green assay
