# Supplementary material for: Feasibility of investigating the association between bacterial pathogens and oral leukoplakia in low and middle income countries: A population-based pilot study in India
Source: PLoS One. 2021 Apr 29;16(4):e0251017. doi: 10.1371/journal.pone.0251017 (PMC8084244; doi:10.1371/journal.pone.0251017)
Supplement: S4 Table — (DOCX) [file pone.0251017.s006.docx]

**S4 Table:** Distribution of *P. gingivalis (Pg)*, *F. nucleatum (Fn)* and *P. intermedia (Pi)* in salivary rinse samples among participants without a clinical diagnosis of oral leukoplakia between 2014 and 2016 by tobacco use status in any form (N=69)

| Characteristics  Number (%) | Never tobacco use  (N= 45) | Ever tobacco use  (N=24) | p-value* |
| --- | --- | --- | --- |
| *Pg* detected** | 45 (100%) | 25 (100%) | - |
| *Pg* quantified | 44 (98%) | 23 (96%) | 0.65 |
| *Pg* copies/ng of DNA, median (IQR) | 1.10X10^4^ (6.01X10^3^, 2.60X10^4^) | 7.24X10^3^ (3.24X10^3^, 2.52X10^4^) | 0.32 |
| *Fn* detected** | 45 (100%) | 23 (96%) | 0.17 |
| *Fn* quantified | 38 (84%) | 19 (79%) | 0.58 |
| *Fn* copies/ng of DNA, median (IQR) | 1.64X10^4^ (8.85X10^3^, 2.99X10^4^) | 1.52X10^4^ (4.86X10^3^, 2.18X10^4^) | 0.50 |
| *Pi* detected*** | 21 (47%) | 14 (58%) | 0.36 |
| *Pi* quantified | 21 (47%) | 13 (54%) | 0.55 |
| *Pi* copies/ng of DNA, median (IQR) | 2.75X10^4^ (1.3X10^4^, 4.42X10^4^) | 2.0X10^4^ (1.38X10^4^, 8.28X10^4^) | 0.71 |
| Any one pathogen detected | 45 (100%) | 24 (100%) | - |
| Any one pathogen quantified | 45 (100%) | 23 (96%) | 0.17 |
| All three pathogens detected | 21 (47%) | 13 (54%) | 0.55 |
| All three pathogens quantified | 20 (44%) | 9 (38%) | 0.58 |
| Total pathogen copies/ng of DNA  median (IQR) | 3.93x10^4^ (1.80x10^4^, 9.00x10^4^) | 2.52x10^4^ (1.56x10^4^, 7.84x10^4^) | 0.58 |

*Chi-square test and Wilcoxon Rank-sum test for differences in proportion and median respectively.

**Taqman assay ***Sybr Green assay
