# Supplementary material for: Feasibility of investigating the association between bacterial pathogens and oral leukoplakia in low and middle income countries: A population-based pilot study in India
Source: PLoS One. 2021 Apr 29;16(4):e0251017. doi: 10.1371/journal.pone.0251017 (PMC8084244; doi:10.1371/journal.pone.0251017)
Supplement: S5 Table — (DOCX) [file pone.0251017.s007.docx]

**S5 Table:** Distribution of *P. gingivalis (Pg)*, *F. nucleatum (Fn)* and *P. intermedia (Pi)* in salivary rinse samples among participants without a clinical diagnosis of oral leukoplakia between 2014 and 2016 by alcohol use status (N=69)

| Characteristics  Number (%) | Never alcohol use  (N= 40) | Ever alcohol use  (N=29) | p-value* |
| --- | --- | --- | --- |
| *Pg* detected** | 40 (100%) | 29 (100%) | - |
| *Pg* quantified | 39 (98%) | 28 (97%) | 0.82 |
| *Pg* copies/ng of DNA, median (IQR) | 1.03X10^4^ (5.93X10^3^, 2.88X10^4^) | 9.57X10^3^ (3.79X10^3^, 2.47X10^4^) | 0.52 |
| *Fn* detected** | 40 (100%) | 28 (97%) | 0.24 |
| *Fn* quantified | 36 (90%) | 21 (72%) | 0.057 |
| *Fn* copies/ng of DNA, median (IQR) | 1.47X10^4^ (6.3X10^3^, 2.25X10^4^) | 1.89X10^4^ (1.12X10^4^, 3.39X10^4^) | 0.15 |
| *Pi* detected*** | 18 (45%) | 17 (59%) | 0.26 |
| *Pi* quantified | 18 (45%) | 16 (55%) | 0.40 |
| *Pi* copies/ng of DNA, median (IQR) | 3.94X10^4^ (1.24X10^4^, 5.13X10^4^) | 2.10X10^4^ (1.42X10^4^, 4.80X10^4^) | 0.65 |
| Any one pathogen detected | 40 (100%) | 29 (100%) | - |
| Any one pathogen quantified | 40 (100%) | 28 (97%) | 0.24 |
| All three pathogens detected | 18 (45%) | 16 (55%) | 0.40 |
| All three pathogens quantified | 17 (43%) | 12 (41%) | 0.93 |
| Total pathogen copies/ng of DNA  median (IQR) | 2.97x10^4^ (1.55x10^4^, 8.67x10^4^) | 3.34x10^4^ (1.88x10^4^, 8.01x10^4^) | 0.66 |

*Chi-square test and Wilcoxon Rank-sum test for differences in proportion and median respectively.

**Taqman assay ***Sybr Green assay
