# Supplementary material for: Feasibility of investigating the association between bacterial pathogens and oral leukoplakia in low and middle income countries: A population-based pilot study in India
Source: PLoS One. 2021 Apr 29;16(4):e0251017. doi: 10.1371/journal.pone.0251017 (PMC8084244; doi:10.1371/journal.pone.0251017)
Supplement: S6 Table — (DOCX) [file pone.0251017.s008.docx]

**S6 Table:** Distribution of *P. gingivalis (Pg)*, *F. nucleatum (Fn)* and *P. intermedia (Pi)* in salivary rinse samples among participants without a clinical diagnosis of oral leukoplakia between 2014 and 2016 by dietary pattern (N=69)

| Characteristics  Number (%) | Non-vegetarian  (N=32) | Vegetarian  (N=37) | p-value* |
| --- | --- | --- | --- |
| *Pg* detected** | 32 (100%) | 37 (100%) | - |
| *Pg* quantified | 31 (97%) | 36 (97%) | 0.96 |
| *Pg* copies/ng of DNA, median (IQR) | 1.17X10^4^ (6.65X10^3^, 3.06X10^4^) | 7.77X10^3^ (3.77X10^3^, 2.32X10^4^) | 0.12 |
| *Fn* detected** | 31 (97%) | 37 (100%) | 0.28 |
| *Fn* quantified | 26 (81%) | 31 (84%) | 0.78 |
| *Fn* copies/ng of DNA, median (IQR) | 1.34X10^4^ (7.38X10^3^, 2.25X10^4^) | 1.72X10^4^ (9.6X10^3^, 2.99X10^4^) | 0.40 |
| *Pi* detected*** | 16 (50%) | 19 (51%) | 0.91 |
| *Pi* quantified | 15 (47%) | 19 (51%) | 0.71 |
| *Pi* copies/ng of DNA, median (IQR) | 2.26X10^4^ (1.30X10^4^, 5.75X10^4^) | 2.75X10^4^ (1.24X10^4^, 5.13X10^4^) | 0.88 |
| Any one pathogen detected | 32 (100%) | 37 (100%) | - |
| Any one pathogen quantified | 31 (97%) | 37 (100%) | 0.28 |
| All three pathogens detected | 15 (47%) | 19 (51%) | 0.71 |
| All three pathogens quantified | 14 (44%) | 15 (41%) | 0.79 |
| Total pathogen copies/ng of DNA  median (IQR) | 3.07 x10^4^ (1.84x10^4^, 9.00x10^4^) | 3.00x10^4^ (1.56x10^4^, 8.35x10^4^) | 0.84 |

*Chi-square test and Wilcoxon Rank-sum test for differences in proportion and median respectively.

**Taqman assay ***Sybr Green assay
