# Supplementary material for: Feasibility of investigating the association between bacterial pathogens and oral leukoplakia in low and middle income countries: A population-based pilot study in India
Source: PLoS One. 2021 Apr 29;16(4):e0251017. doi: 10.1371/journal.pone.0251017 (PMC8084244; doi:10.1371/journal.pone.0251017)
Supplement: S7 Table — (DOCX) [file pone.0251017.s009.docx]

**S7 Table:** Distribution of *P. gingivalis (Pg)*, *F. nucleatum (Fn)* and *P. intermedia (Pi)* in salivary rinse samples among participants without a clinical diagnosis of oral leukoplakia between 2014 and 2016 by periodontal disease status (N=69)

| Characteristics  Number (%) | No periodontal disease  (N=46) | Clinically diagnosed periodontal disease (N=23) | p-value* |
| --- | --- | --- | --- |
| *Pg* detected** | 46 (100%) | 23 (100%) | - |
| *Pg* quantified | 45 (98%) | 22 (96%) | 0.61 |
| *Pg* copies/ng of DNA, median (IQR) | 9.45X10^3^ (4.3X10^3^, 2.43X10^4^) | 9.97X10^3^ (6.03 X10^3^, 2.74X10^4^) | 0.50 |
| *Fn* detected** | 46 (100%) | 22 (96%) | 0.16 |
| *Fn* quantified | 38 (83%) | 19 (83%) | 0.99 |
| *Fn* copies/ng of DNA, median (IQR) | 1.44X10^4^ (7.65X10^3^, 2.67X10^4^) | 1.76X10^4^ (1.17X10^4^, 3.19X10^4^) | 0.47 |
| *Pi* detected*** | 21 (46%) | 14 (61%) | 0.23 |
| *Pi* quantified | 21 (46%) | 13 (57%) | 0.39 |
| *Pi* copies/ng of DNA, median (IQR) | 2.20X10^4^ (1.24X10^4^, 4.60X10^4^) | 3.53X10^4^ (1.65X10^4^, 8.28X10^4^) | 0.35 |
| Any one pathogen detected | 46 (100%) | 23 (100%) | - |
| Any one pathogen quantified | 46 (100%) | 22 (96%) | 0.15 |
| All three pathogens detected | 21 (46%) | 13 (57%) | 0.39 |
| All three pathogens quantified | 18 (39%) | 11 (48%) | 0.49 |
| Total pathogen copies/ng of DNA  median (IQR) | 2.72x10^4^ (1.56x10^4^, 7.26x10^4^) | 4.69x10^4^ (2.10x10^4^, 9.70x10^4^) | 0.20 |

*Chi-square test and Wilcoxon Rank-sum test for differences in proportion and median respectively.

**Taqman assay ***Sybr Green assay
